# Optimal Postoperative Treatment for Composite Laryngeal Small Cell Carcinoma

**DOI:** 10.1155/2013/806284

**Published:** 2013-09-15

**Authors:** Koji Ebisumoto, Akihiro Sakai, Kenji Okami, Ryousuke Sugimoto, Kosuke Saito, Masahiro Iida

**Affiliations:** Department of Otolaryngology-Head and Neck Surgery, Tokai University, School of Medicine, Isehara 259-1193, Japan

## Abstract

Small cell carcinoma (SmCC) most commonly occurs in the lung and rarely arises from the head and neck region. Further, composite SmCC is extremely rare. Therefore, no postoperative treatment strategy has been established. We report a 59-year-old male patient referred to our outpatient clinic for further examination and treatment of a laryngeal tumor. Biopsy from the tumor revealed squamous cell carcinoma (SCC). The preoperative diagnosis was supraglottic SCC (T3N2bM0), and total laryngectomy and bilateral neck dissection were performed. Pathological examination revealed 2 individual cancer components: SmCC and SCC. Postoperative chemoradiotherapy (2 courses of cisplatin (CDDP) and etoposide (VP-16)) was indicated. Following the postoperative chemoradiotherapy, 2 courses of adjuvant chemotherapy were administered. The patient is currently alive with no evidence of disease at 36 months following the completion of therapy. Postoperative chemoradiotherapy and adjuvant chemotherapy are optimal treatment strategies for laryngeal composite SmCC.

## 1. Introduction

Small cell carcinoma (SmCC) most commonly occurs in the lung and rarely arises from the head and neck region. SmCC combined with another carcinoma is classified as combined or composite SmCC and is extremely rare. SmCC arising in the larynx accounts for only 0.5% of all laryngeal cancers, and composite SmCC arising in the larynx accounts for less than 10% of laryngeal SmCC [1]. Therefore, clinical experiences are very limited. The standard treatment for laryngeal and laryngeal composite SmCC is chemotherapy and radiotherapy [2–4]. However, it is difficult to diagnose laryngeal composite SmCC before an operation [5]. For these patients, systemic postoperative treatment is essential. However, no postoperative treatment strategy for composite SmCC in the head and neck region has been established. Here, we present a case of laryngeal composite SmCC and discuss the optimal postoperative treatment.

## 2. Case Presentation

A 59-year-old male patient was referred to our outpatient clinic for further examination and treatment of a laryngeal tumor. The patient had no remarkable medical or family history, was a heavy smoker (60 pack-years), and was a social drinker. 

**Figure 1:**
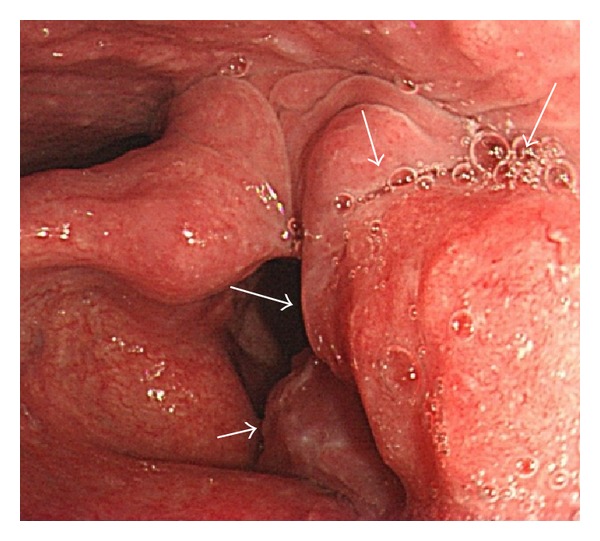
Preoperative findings of the larynx. The tumor was seen at the left arytenoid (arrow), and the left vocal cord was fixed.

The tumor was detected at the left arytenoid, and the left vocal cord was fixed (Figure 1). A computed tomography (CT) scan showed that the thyroid cartilage was intact. Several cervical lymph node metastases were detected on the left side of the neck; however, distant metastasis was not detected. Cervical ultrasonography also revealed multiple metastatic lymph nodes on the left side. Squamous cell carcinoma (SCC) was diagnosed from the biopsy specimen of the primary site. The preoperative diagnosis was supraglottic cancer (T3N2bM0), Stage IVA. 

**Figure 2:**
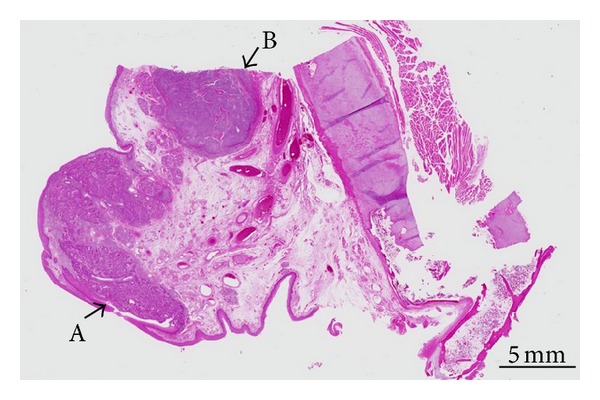
Loupe image of the larynx. Two individual cancer components were noted: A (A) squamous cell carcinoma (SCC) component and a (B) small cell carcinoma (SmCC) component. Collision was not seen among these 2 components.

**Figure 3:**
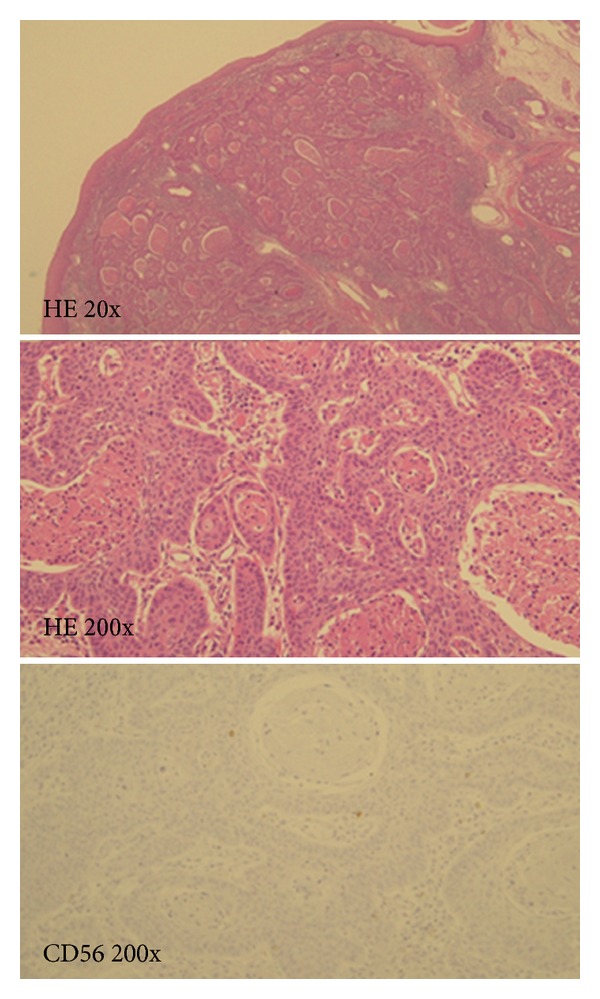
The SCC component of the tumor. The first component was highly differentiated SCC with a cancer pearl. CD56 was not expressed.

**Figure 4:**
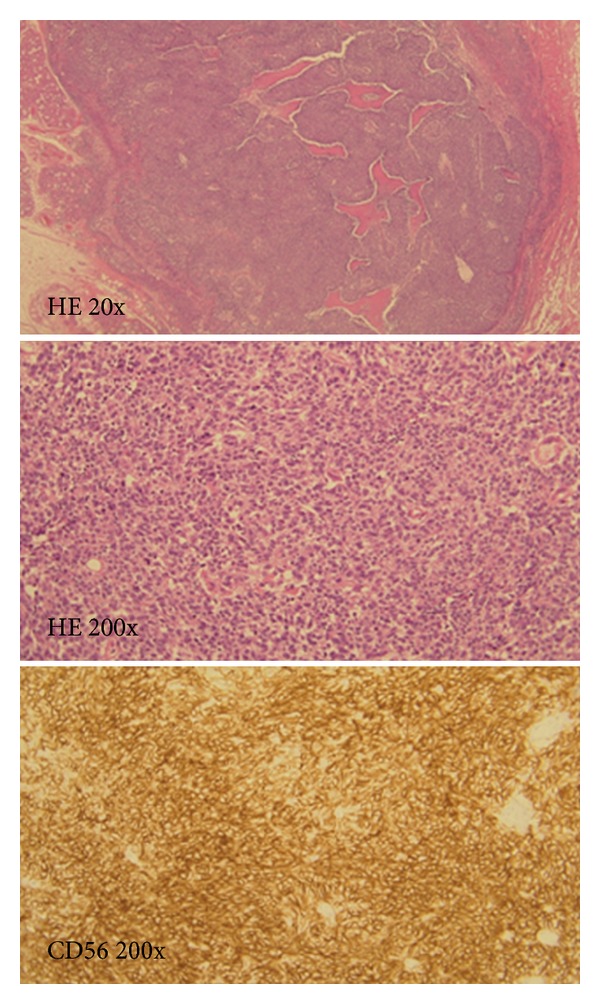
The SmCC component of the tumor. The second component was composed of small round cancer cells, and CD56 was highly expressed.

Total laryngectomy and bilateral neck dissection were performed. Pathological examination of the larynx indicated 2 individual cancer components that were histologically separate from each other (Figure 2). The first component was highly differentiated SCC with a cancer pearl, and CD56 was not expressed (Figure 3). The second component was SmCC with highly expressed CD56 (Figure 4). Four lymph node metastases of SCC without extracapsular spread were found in the left neck.

Postoperative radiotherapy (50 Gy) with 2 concurrent courses of chemotherapy (80 mg/m^2^ cisplatin (CDDP) and 100 mg/m^2^ etoposide (VP-16)) was indicated. Following postoperative chemoradiotherapy, 2 courses of adjuvant chemotherapy with the same regimen were administered. The patient is currently alive with no evidence of disease at 36 months following the completion of the treatment.

## 3. Discussion

SmCC arising in the larynx accounts for 0.5% of all laryngeal cancers. SmCC combined with another carcinoma, such as SCC or adenocarcinoma, is classified as combined or composite SmCC. Composite SmCC is extremely rare and accounts for less than 10% of laryngeal small cell carcinomas [1].

The prognosis of laryngeal SmCC is worse than that of laryngeal SCC because of its aggressive characteristics. SmCC grows rapidly and easily results in lymph node and distant metastasis. The 2- and 5-year survival rates are 16% and 5%, respectively [2, 5, 6]. The prognosis of composite SmCC is the same as that of SmCC, and the vast majority of patients die within 2 years [3, 5].

According to Aggarwal et al., preoperative diagnosis of composite tumors is difficult, and detailed examination of the operation specimen is important [5]. Laryngeal cancer is usually diagnosed by biopsy under local anesthesia in an outpatient setting. Biopsy under general anesthesia is performed for a limited number of patients who cannot undergo biopsy under local anesthesia. Determination of whether the tumor is a composite SmCC is impossible using the biopsy specimen. Given its rarity, the diagnosis of composite SmCC depends on the operation specimen.

The treatment for composite SmCC is the same as that for SmCC. Surgical treatment, such as total laryngectomy, is one of the treatment strategies. However, the standard treatment for SmCC is chemotherapy and radiotherapy because of its extremely poor prognosis [2–4]. The prognosis of SmCC is not related to tumor size [6], and the operation or postoperative irradiation cannot improve the prognosis [2]. Although the regimen for chemotherapy is not well established, CDDP and VP-16 may improve the prognosis. Systemic chemotherapy is essential throughout the course of treatment; however, the suitable period for chemotherapy, that is, neoadjuvant, concurrent, or adjuvant, is controversial [2].

In our case, the patient had 4 lymph node metastases of SCC; therefore, postoperative irradiation was required. As stated above, chemotherapy is essential for SmCC treatment. According to the report by Bardeaux et al., adjuvant chemotherapy may improve the prognosis; therefore, the patient was treated with adjuvant chemotherapy after postoperative chemoradiotherapy.

SmCC occurs more frequently in the lung. For lung SmCC patients, surgical treatment is limited, and postoperative treatment with systemic chemotherapy is essential [7]. Thoracic radiotherapy for limited types of SmCC improves local control; however, chemoradiotherapy provides a 5% better prognosis than radiotherapy alone [8]. In the National Comprehensive Cancer Network guidelines, chemoradiotherapy is recommended for cases with lymph node metastasis, whereas chemotherapy is recommended for cases without lymph node metastasis [9].

Although lung SmCC treatments cannot be directly adapted to head and neck SmCC, the knowledge of lung treatments suggests that postoperative treatment is necessary for laryngeal composite SmCC patients. We conclude that postoperative chemoradiotherapy with adjuvant chemotherapy may be one of the optimal treatments for laryngeal composite SmCC patients.
